# Super High-k Dielectric via Composition-Dependent Hafnium Zirconium Oxide Superlattice for Si Nanosheet Gate-All-Around Field-Effect Transistors with NH_3_ Plasma-Optimized Interfaces

**DOI:** 10.3390/ma18081740

**Published:** 2025-04-10

**Authors:** Yi-Ju Yao, Yu-Min Fu, Yu-Hung Chen, Chen-You Wei, Kai-Ting Huang, Guang-Li Luo, Fu-Ju Hou, Yu-Sheng Lai, Yung-Chun Wu

**Affiliations:** 1 College of Semiconductor Research, National Tsing Hua University, Hsinchu 30013, Taiwan; 2 Department of Engineering and System Science, National Tsing Hua University, Hsinchu 30013, Taiwan; 3 Taiwan Semiconductor Research Institute, Hsinchu 30078, Taiwan

**Keywords:** high-k, k-value, EOT, hafnium oxide, zirconium oxide, nanosheet, GAAFET, Si, superlattice, NH_3_ plasma, interfacial layers

## Abstract

This paper presents an advanced dielectric engineering approach utilizing a composition-dependent hafnium zirconium oxide (Hf_1-x_Zr_x_O_2_) superlattice (SL) structure for Si nanosheet gate-all-around field-effect transistors (Si NSGAAFETs). The dielectric (DE) properties of solid solution (SS) and SL Hf_1-x_Zr_x_O_2_ capacitors were systematically characterized through capacitance-voltage (C-V) and polarization-voltage (P-V) measurements under varying annealing conditions. A high dielectric constant (k-value) of 59 was achieved in SL-Hf_0.3_Zr_0.7_O_2_, leading to a substantial reduction in equivalent oxide thickness (EOT). Furthermore, the SL-Hf_0.3_Zr_0.7_O_2_ dielectric was integrated into Si NSGAAFETs, with the interfacial layer (IL) further optimized via NH_3_ plasma treatment. The resulting devices exhibited superior electrical performance, including an enhanced ON-OFF current ratio (I_ON_/I_OFF_) reaching 10^7^, an increased drive current, and significantly reduced gate leakage. These results highlight the potential of SL-Hf_0.3_Zr_0.7_O_2_ as a high-k dielectric solution for overcoming EOT scaling challenges in advanced CMOS technology and enabling further innovation in next-generation logic applications.

## 1. Introduction

As device scaling progresses, maintaining Moore’s Law becomes increasingly challenging, particularly for logic applications at the 2 nm node and beyond [1,2]. A key obstacle lies in reducing the equivalent oxide thickness (EOT) while simultaneously enhancing the drive current to meet stringent performance and miniaturization demands [3,4]. In this regard, ferroelectric (FE) materials such as HfO_2_-ZrO_2_ (HZO) have attracted significant attention due to their higher dielectric constant (k-value) compared to conventional HfO_2_, enabling substantial EOT scaling [5,6,7]. The k-value for HfO_2_ typically ranges from 16 to 20, while FE materials like HZO can achieve k-values in the range of 25 to 40. Studies have shown that increasing the ZrO_2_ content in SS-HZO further enhances the k-value [8,9,10]. However, the intrinsic hysteresis effects of ferroelectric materials can induce threshold voltage (V_TH_) shifts, complicating their integration into logic transistors [11,12].

To address these challenges, recent advancements in high-k dielectrics—particularly HfO_2_/ZrO_2_ SL—have demonstrated significant potential [13,14,15]. The enhanced k-value in SL structures originates from the morphotropic phase boundary (MPB) between orthorhombic(o-phase) and tetragonal (t-phase) phases, which not only improve the dielectric constant but also mitigate hysteresis and leakage current, making them highly suitable for advanced semiconductor devices [16,17].

In this work, we propose an optimized SL-Hf_0.3_Zr_0.7_O_2_ dielectric for Si NSGAAFETs targeting the 2 nm technology node. The optimization process involves a systematic investigation of key parameters, including the selection of the Zr molar fraction in the HfO_2_-ZrO_2_ system and a comparative analysis between SL and SS structures. This approach allowed us to identify the optimal conditions for achieving the highest k-value and the best overall device performance. The Hf_1-x_Zr_x_O_2_ dielectric properties of capacitors were systematically analyzed through capacitance-voltage (C-V) and polarization-voltage (P-V) measurements, with SS and SL configurations compared across varying annealing temperatures. The proposed SL-Hf_0.3_Zr_0.7_O_2_ dielectric was then integrated into Si NSGAAFETs, with the IL further optimized via NH_3_ plasma treatment. The resulting devices exhibited superior electrical performance, including an improved I_ON_/I_OFF_ ratio, an increased drive current, and a significantly reduced leakage current. These findings highlight the potential of SL-Hf_0.3_Zr_0.7_O_2_ in overcoming critical challenges in CMOS scaling, paving the way for further innovations in next-generation logic applications.

## 2. Device Fabrication

Initially, the single-crystal Si layer on the SOI wafer was thinned down to 25 nm. The Ge/Si/Ge/Si/Ge SL layers were then epitaxially grown using low-pressure chemical vapor deposition (LPCVD), with each layer approximately 25 nm thick. The active region of the device was defined via electron beam lithography (EBL), followed by patterning through reactive ion etching (RIE) as isotropic etching gases Cl_2_ for downward etching. After cleaning, the Ge sacrificial layers were selectively removed using an H_2_O_2_-based solution, enabling the release of the nanosheet (NS) channel. The Si NS had a thickness of 25 nm each, and the channel direction was oriented along the <110> crystallographic direction. The Si layer was grown without additional doping during the epitaxy process.

The wafer was transferred to an atomic layer deposition (ALD) chamber, where the native IL underwent NH_3_ plasma treatment to enhance interface quality and reduce defect formation. This treatment was performed at 250 °C with a power setting of 200 watts for 120 s, utilizing ammonia gas. Subsequently, a 5 nm thick SL-Hf_0.3_Zr_0.7_O_2_ dielectric was deposited via ALD, consisting of alternating 0.3 nm and 0.7 nm layers. The Hf_1-x_Zr_x_O_2_ structure was engineered using a cycle-controlled layer-by-layer deposition process to ensure precise composition modulation. Gate metal deposition was then carried out using a combination of ALD and physical vapor deposition (PVD) to ensure full NS gate encapsulation.

The gate pattern was subsequently transferred onto the substrate through EBL and RIE (also with isotropic etching gases Cl_2_). Following gate formation, the source/drain (S/D) regions were doped with n-type impurities using phosphorus ion implantation at doses of 1 × 10^15^ cm^−2^, with implantation energies of 10 keV, 20 keV, and 30 keV to achieve optimized doping profiles. Finally, dielectric crystallization and dopant activation were performed in a nitrogen ambient using rapid thermal annealing (RTA) at 550 °C for 30 s. This systematic fabrication process ensures the realization of high-performance Si NSGAAFET devices with the optimized SL-Hf_0.3_Zr_0.7_O_2_ dielectric, demonstrating its feasibility for advanced CMOS scaling.

**Figure 1:**
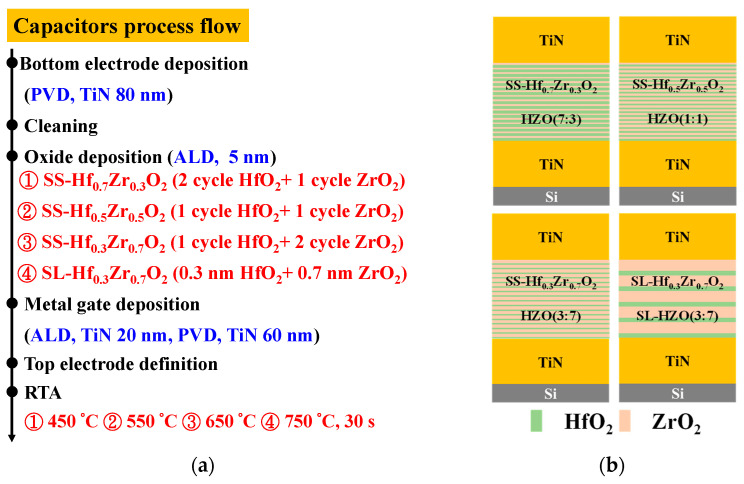
(**a**) Fabrication process flow and (**b**) cross-sectional schematic of Hf_1-x_Zr_x_O_2_ MIM capacitors with SS and SL configurations.

**Figure 2:**
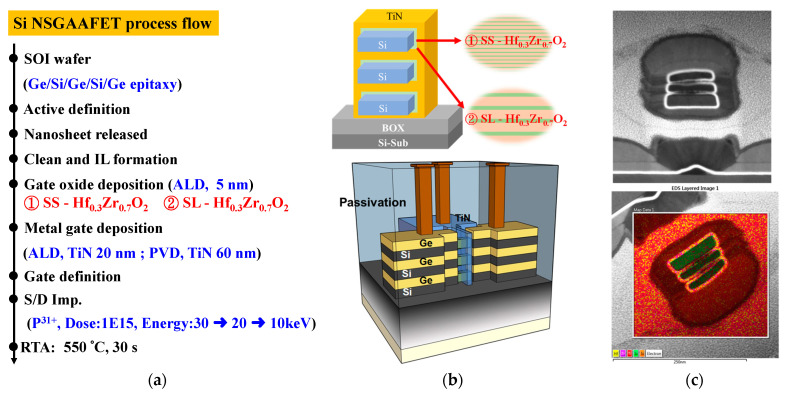
(**a**) Process flow and (**b**) cross-sectional schematic of Si NSGAAFET. (**c**) TEM image and EDS mapping of Si NSGAAFET, showing devices with a final high-k stack of 5.13 nm and NS structure.

Figure 1a illustrates the process flow of capacitor fabrication, while Figure 1b shows the schematic cross-sectional view of the Hf_1-x_Zr_x_O_2_ metal-insulator-metal (MIM) capacitors with SS and SL configurations. Figure 2a illustrates the Si NSGAAFETs process flow, while Figure 2b shows the schematic cross-sectional view of the Si NSGAAFET, clearly depicting the structural configuration of the device. Figure 2c presents the transmission electron microscope (TEM) image and energy-dispersive X-ray spectroscopy (EDS) mapping of the Si NSGAAFET. The high-k dielectric layer, consisting of cycle-controlled SS-Hf_0.3_Zr_0.7_O_2_ and SL-Hf_0.3_Zr_0.7_O_2_, features individual layer thicknesses of 0.3 nm for HfO_2_ and 0.7 nm for ZrO_2_, resulting in a final stack thickness of nearly 5.13 nm.

## 3. Results and Discussion

### 3.1. Investigation of Composition-Dependent Hafnium Zirconium Oxide Capacitors with Solid Solution and Superlattice Structure

**Figure 3:**
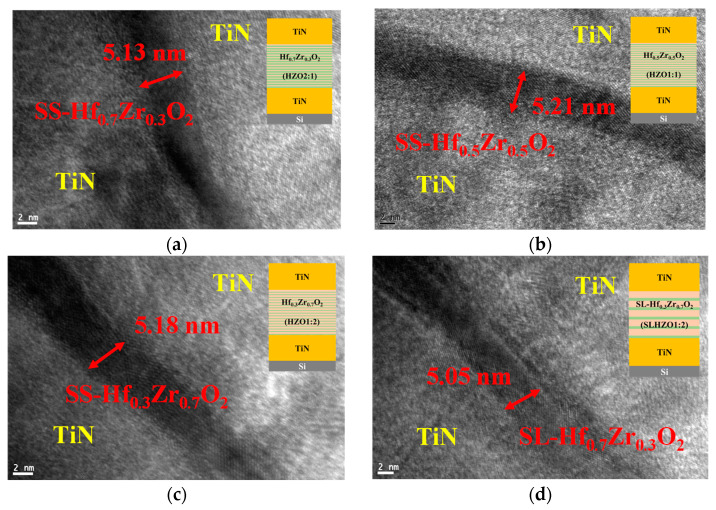
Cross-sectional TEM images of MIM capacitors with approximately 5 nm thick (**a**) SS-Hf_0.7_Zr_0.3_O_2_; (**b**) SS-Hf_0.5_Zr_0.5_O_2_; (**c**) SS-Hf_0.3_Zr_0.7_O_2_; and (**d**) SL-Hf_0.3_Zr_0.7_O_2_ dielectric layers. The thickness of dielectric layers and materials as shown in the figures.

Figure 3a–d show the transmission electron microscope (TEM) images of the four conditions: SS-Hf_0.7_Zr_0.3_O_2_, SS-Hf_0.5_Zr_0.5_O_2_, SS-Hf_0.3_Zr_0.7_O_2_, and SL-Hf_0.3_Zr_0.7_O_2_ MIM capacitors, respectively, had a total thickness of approximately 5 nm. All devices in this study were fabricated and tested alongside control samples to ensure consistency and mitigate potential variations.

**Figure 4:**
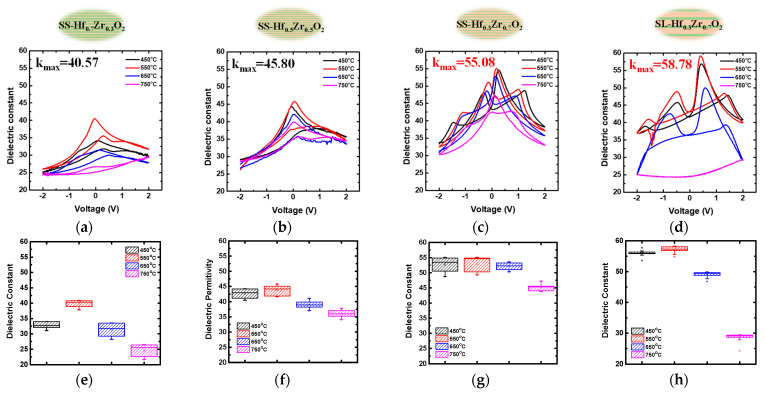
Temperature-dependent dielectric constant versus voltage characteristics of Hf_1-x_Zr_x_O_2_ MIM capacitors: (**a**–**d**) k-V curves and (**e**–**h**) corresponding k-value distribution box plots for (**a**,**e**) SS-Hf_0.7_Zr_0.3_O_2_, (**b**,**f**) SS-Hf_0.5_Zr_0.5_O_2_, (**c**,**g**) SS-Hf_0.3_Zr_0.7_O_2_, and (**d**,**h**) SL-Hf_0.3_Zr_0.7_O_2_. The SL-Hf_0.3_Zr_0.7_O_2_ exhibits an enhanced k-value (~59) at 550 °C due to MPB-induced o-phase and t-phase coexistence.

Figure 4a–d present the dielectric constant versus voltage characteristics calculated from the C-V curves of MIM capacitors incorporating Hf_1-x_Zr_x_O_2_ dielectrics with both SS and SL structures at different temperatures. The SS-Hf_0.7_Zr_0.3_O_2_ dielectric exhibits behavior similar to conventional HfO_2_ dielectrics, while SS-Hf_0.5_Zr_0.5_O_2_ demonstrates characteristics typical of ferroelectric materials, with a peak capacitance near 0 V due to dipole polarization. Corresponding box plots of k-values further validate these findings in Figure 4e–h, respectively. The SS-Hf_0.3_Zr_0.7_O_2_ dielectric shows a high k-value of 55 at 550 °C, which is primarily attributed to the morphotropic phase boundary (MPB) effect, where the coexistence of the o-phase and t-phase enhances dielectric performance [18]. As the ZrO_2_ content increases, the observed shift in the peak k-value with temperature suggests a phase transition between the o-phase and t-phase [19]. This boundary not only improves the dielectric constant but also reduces the hysteresis and leakage current, making SL structures highly suitable for advanced semiconductor devices. Furthermore, the statistical analysis of the phase diagrams shows that the distribution in Figure 4h is denser than that in Figure 4g, confirming that the presence of the MPB enhances the stability of the dielectric layer.

When utilizing the SL stacking structure, the SL-Hf_0.3_Zr_0.7_O_2_ dielectric achieves a higher k-value of 59 at 550 °C, and the statistical data show a more concentrated and stable distribution. These results indicate that the SL stacking method significantly enhances both the k-value and electrical stability compared to the SS approach. This improvement is attributed to the mechanical stress and changes in free energy at the interfaces, which promote superior electrical performance. However, at high annealing temperatures of 750 °C, there is a significant decrease in both the dielectric constant. The capacitance values markedly decrease, leading to a noticeable reduction in the k-value.

**Figure 5:**
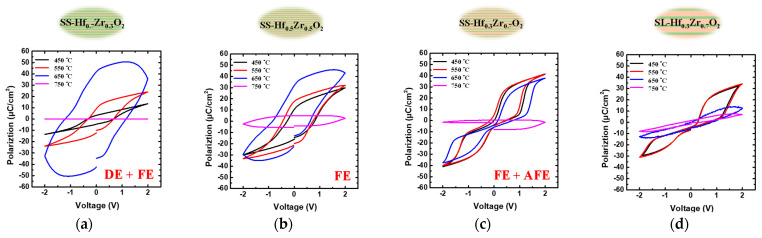
Temperature-dependent P-V characteristics of Hf_1-x_Zr_x_O_2_ MIM capacitors with (**a**) SS-Hf_0.7_Zr_0.3_O_2_ showing mixed DE/FE phases with 2Pr ~20 µC/cm^2^; (**b**) SS-Hf_0.5_Zr_0.5_O_2_ exhibiting ferroelectric behavior with 2Pr ~40 µC/cm^2^; (**c**) SS-Hf_0.3_Zr_0.7_O_2_; and (**d**) SL-Hf_0.3_Zr_0.7_O_2_ demonstrating FE/AFE phase coexistence with 2Pr ~10 µC/cm^2^ and 0 µC/cm^2^.

Figure 5a–d present the P-V characteristics of MIM structures incorporating Hf_1-x_Zr_x_O_2_ dielectrics at various annealing temperatures. The SS-Hf_0.7_Zr_0.3_O_2_ film demonstrates a combination of dielectric and ferroelectric phases, particularly at 450 °C. The SS-Hf_0.7_Zr_0.3_O_2_ sample exhibits a reduced hysteresis loop with a remanent polarization (2Pr) of approximately 20 µC/cm^2^. In contrast, the SS-Hf_0.5_Zr_0.5_O_2_ sample displays a typical ferroelectric hysteresis loop with a 2Pr value of around 40 µC/cm^2^, making it suitable for ferroelectric memory applications. Both the SS-Hf_0.3_Zr_0.7_O_2_ and SL-Hf_0.3_Zr_0.7_O_2_ samples exhibit a combination of ferroelectric and antiferroelectric (AFE) phases, with a 2Pr value of approximately 10 µC/cm^2^ and 0 µC/cm^2^ near 0 V. The primary challenge with FE logic devices lies in the internal electric fields generated by dipoles, which lead to hysteresis effects. This hysteresis can cause significant shifts in the V_TH_, making these devices less suitable for logic applications where precise control over switching is critical. The proposed characteristics of the capacitance can effectively reduce V_TH_ shift, making it suitable for logic device applications. Notably, the SL-Hf_0.3_Zr_0.7_O_2_ dielectric shows minimal leakage current as the temperature increases, indicating the structural stability of this material [20]. The residual polarization decreases significantly at this elevated temperature, indicating that high thermal conditions adversely affect the dielectric properties of the material. These findings further correlate with the peak capacitance observed in Figure 4, where the position of the capacitance peak is attributed to the interplay between the different phases.

### 3.2. Comparison and Enhancement of Silicon Nanosheet Gate-All-Around Field-Effect Transistors with Solid Solution and Superlattice Structures

**Figure 6:**
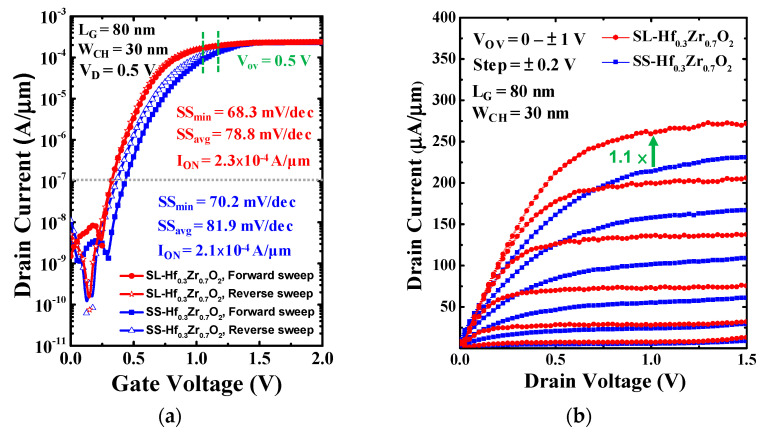
(**a**) I_D_-V_G_ and (**b**) I_D_-V_D_ characteristics of N-type Si NSGAAFETs (W_CH_ = 30 nm, L_G_ = 80 nm) with SS and SL-Hf_0.3_Zr_0.7_O_2_ forward (solid) and reverse (dashed) sweeps. The SL structure exhibits improved performance compared to the SS structure.

Figure 6a demonstrates the drain current (I_D_) versus gate voltage (V_G_), and Figure 6b demonstrates the I_D_ versus drain voltage (V_D_) characteristics of N-type Si NSGAAFETs incorporating SS-Hf_0.3_Zr_0.7_O_2_ and SL-Hf_0.3_Zr_0.7_O_2_ dielectrics. The device dimensions include a channel width (W_CH_) of 30 nm and a gate length (L_G_) of 80 nm. Both forward (solid line) and reverse (dashed line) sweeps are shown, confirming minimal hysteresis. Compared to the SS-Hf_0.3_Zr_0.7_O_2_ dielectric, the SL structure significantly reduces hysteresis while maintaining a higher I_ON_. Specifically, the SS-Hf_0.3_Zr_0.7_O_2_ sample exhibits an average subthreshold swing (SS_avg_ = 81.9 mV/dec), a minimum subthreshold swing (SS_min_ = 70.2 mV/dec), and I_ON_ of 2.1 × 10^−4^. In contrast, the SL-Hf_0.3_Zr_0.7_O_2_ sample shows an average subthreshold swing (SS_avg_ = 78.8 mV/dec), minimum subthreshold swing (SS_min_ = 68.3 mV/dec), and I_ON_ of 2.3 × 10^−4^, further confirming the enhanced performance. However, the off-current reached a relatively large value of 10^−9^ A/µm at V_G_ = 0 and V_D_ = 0.5 V. This significant off-current may be attributed to poor interface quality, which can lead to enhanced leakage effects. Defects at the interface can create additional pathways for current flow, resulting in increased off-current levels in the device. The normalized I_D_, divided by the footprint width and V_TH_, was determined at a constant I_D_ of 10^−7^ A/µm.

**Figure 7:**
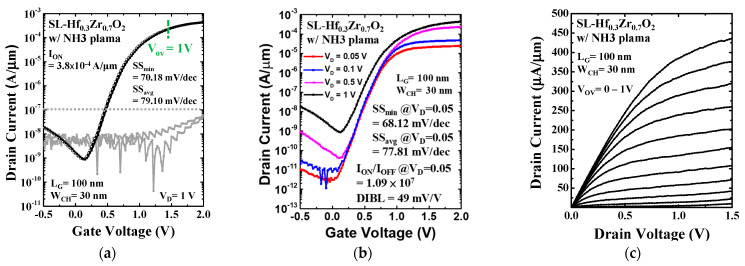
(**a**) I_D_-V_G_ characteristics of the SL-Hf_0.3_Zr_0.7_O_2_ dielectric applied on N-type Si NSGAAFETs with NH_3_ plasma treatment showing reduced I_G_ and high I_ON_. (**b**) I_D_-V_G_ characteristics with different drain voltage levels, exhibiting enhanced I_ON_/I_OFF_ ratios (~10^7^) with (**c**) corresponding I_D_-V_D_ curves.

To further suppress I_OFF_ in NMOS devices, NH_3_ plasma treatment was employed to enhance the quality of the IL. As shown in Figure 7a, the electrical characteristics of devices treated with NH_3_ plasma show that SS_avg_ = 79.10 mV/dec and SS_min_ = 70.18 mV/dec with an I_ON_ of 3.8 × 10^−4^ and the gate leakage current (I_G_) (shown by the gray curve). Figure 7b shows an improvement in I_OFF_ (shown by a purple curve) and showcases leakage currents as low as 10^−10^A/μm at V_G_ = 0 and V_D_ = 0.5 V. An I_ON_/I_OFF_ of up to 10^7^, SS_avg_ = 77.81 mV/dec, SS_min_ = 68.12 mV/dec, and drain-induced barrier-lowering (DIBL = 49 mV/V) at V_D_ = 0.5 V alongside the I_D_-V_D_ characteristics are demonstrated in Figure 7c. This enhancement underscores the effectiveness of NH_3_ plasma treatment in improving the electrical performance of the devices, making this approach highly compatible with CMOS technology platforms.

Table 1 highlights the performance metrics in comparison with other contemporary devices reported in the literature. Key parameters such as on-off current ratios, subthreshold slopes, and overall device stability are systematically compared. This approach allows for a clearer understanding of the proposed device standards relative to existing technologies.

## 4. Conclusions

This study introduces an optimized SL-Hf_0.3_Zr_0.7_O_2_ dielectric for Si NSGAAFET_S_, demonstrating its potential to address challenges associated with CMOS scaling and advanced logic applications. The dielectric properties of Hf_1-x_Zr_x_O_2_ with SS and SL structures were systematically investigated and compared through C-V and P-V measurements under various annealing conditions. The optimized composition achieved a high dielectric constant of 59, leading to a significant reduction in EOT. When integrated into Si NSGAAFETs, the SL dielectric combined this with the NH_3_ plasma treatment of the IL and exhibited superior electrical performance, including a higher I_ON_/I_OFF_, enhanced drive current, and notably reduced leakage current. These results validate the effectiveness of the SL-Hf_0.3_Zr_0.7_O_2_ dielectric in enhancing the performance of next-generation logic devices.

## Figures and Tables

**Table 1:** Comparison with other contemporary devices.

	This Work	Intel [20]	IBM [21]	Imec [22]
**Structure**	**Si NSGAAFET**	Si NSGAAFET	Si NSGAAFET	Si NSGAAFET
**L_G_ (nm)**	**100**	180	36	18
**|V_D_| (V)**	**0.5**	0.65	0.65	0.7
**SS (mV/dec)**	**69.1**	67	68	72
**DIBL (mV/V)**	**15**	10	27	N/A
**I_ON_/I_OFF_**	**~10^7^**	N/A	~10^6^	~10^6^

## Data Availability

The data presented in this study are available on request from the corresponding author.
